# Changes in the prevalence and biofilm formation of *Haemophilus influenzae* and *Haemophilus parainfluenzae* from the respiratory microbiota of patients with sarcoidosis

**DOI:** 10.1186/s12879-016-1793-7

**Published:** 2016-08-26

**Authors:** Urszula Kosikowska, Paweł Rybojad, Dagmara Stępień–Pyśniak, Anna Żbikowska, Anna Malm

**Affiliations:** 1 Department of Pharmaceutical Microbiology with Laboratory for Microbiological Diagnostics, Medical University of Lublin, Chodzki Str. 1, 20-093 Lublin, Poland; 2 Department of Thoracic Surgery, Medical University of Lublin, Lublin, Poland; 3 Sub-Department of Veterinary Prevention and Avian Diseases, Institute of Biological Bases of Animal Diseases, Faculty of Veterinary Medicine, University of Life Sciences in Lublin, Lublin, Poland; 4 Department of Food Technology, Faculty of Food Sciences, Warsaw University of Life Sciences (WULS-SGGW), Warsaw, Poland

**Keywords:** Sarcoidosis, Respiratory microbiota, *Haemophilus parainfluenzae*, *Haemophilus influenzae*, Biofilm

## Abstract

**Background:**

Healthy condition and chronic diseases may be associated with microbiota composition and its properties. The prevalence of respiratory haemophili with respect to their phenotypes including the ability to biofilm formation in patients with sarcoidosis was assayed.

**Methods:**

Nasopharynx and sputum specimens were taken in 31 patients with sarcoidosis (average age 42.6 ± 13), and nasopharynx specimens were taken in 37 healthy people (average age 44.6 ± 11.6). Haemophili were identified by API-NH microtest and by the matrix-assisted laser desorption/ionization time-of-flight mass spectrometry (MALDI-TOF MS) system. Biofilm was visualised by crystal violet staining and confocal scanning laser microscopy (CSLM). The statistical analysis was performed with Statgraphics Plus for Windows.

**Results:**

In total, 30/31 patients with sarcoidosis and 31/37 healthy people were colonized by *Haemophilus influenzae* (6/30 vs. 1/31) and *Haemophilus parainfluenzae* (28/30 vs. 31/31) in the nasopharynx. The overall number of nasopharyngeal haemophili isolates was 59 in patients with sarcoidosis and 67 in healthy volunteers (*H. influenzae* 6/59 vs. 1/67, *P =* 0.05; *H. parainfluenzae* 47/59 vs. 65/67, *P =* 0.0032). Moreover, the decreased number of *H. parainfluenzae* biofilm-producing isolates was shown in nasopharyngeal samples in patients with sarcoidosis as compared to healthy people (19/31 vs. 57/65, *P =* 0.006), especially with respect to isolates classified as strong and very strong biofilm-producers (8/31 vs. 39/65, *P =* 0.002).

**Conclusions:**

The obtained data suggest that the qualitative and quantitative changes within the respiratory microbiota concerning the overall prevalence of *H. influenzae* together with the decreased number of *H. parainfluenzae* strains and the decreased rate of *H. parainfluenzae* biofilm-producing isolates as compared to healthy people may be associated with sarcoidosis.

## Background

Sarcoidosis is a chronic and enigmatic multisystem disease involving the lung, heart and the lymphatic system [1, 2]. The etiology of sarcoidosis is not likely to be due to any infections, but rather to an exaggerated and aberrant immune response of genetically susceptible individuals to unidentified antigens, including microorganisms, or several organic and inorganic substances [1–4].

Understanding the role of microbiota composition is a new frontier of human biology and the contemporary direction in the investigation of physiological or pathological phenomena of health or diseases [5–7]. Qualitative and quantitative shifts or perturbation in the microbiota can lead to the development of diseases. Microbiota monitoring and modification may be useful for determination of health, thus providing new means of protection and/or of intervention, and data interpretation [8–10]. The microbiota components predominantly colonizing the respiratory mucosa without causing any disease symptoms can occasionally cause respiratory infections. Besides possible positive or negative interactions between commensals and pathobionts as well as other potential pathogens, microbiota can play an important role for the human host organism in preventing of respiratory and invasive infections [11]. Additionally, microbiota disturbance can contribute to acquisition and carriage of pathogens, can predispose to viral co-infection, especially in people with an immature or damaged immune system.

The human-restricted respiratory tract microbiota representatives are *Haemophilus influenzae* with significant pathogenicity and opportunistic commensal *H. parainfluenzae* [12, 13]. They may be etiologic agents of invasive or opportunistic diseases [14–16]. *H. influenzae*, both the encapsulated (mainly serotype b – Hib) and non-encapsulated (nontypeable *H. influenzae* - NTHi) strains have also been associated as potential pathogens with chronic or recurrent and invasive diseases (e.g. bacteremia or sepsis, otitis media, chronic bronchitis, and community-acquired pneumonia) often reported in children and rarely in adults. *H. parainfluenzae*, as an opportunistic bacteria, less often reported as an etiologic agent of infectious diseases, may cause systemic or other respiratory infections (e.g. epiglottitis, meningitis, bacteremia or sepsis, bronchitis, chronic obstructive pulmonary disease, and infective endocarditis).

The human microbiota is a reservoir of opportunistic and potential pathogens (pathobionts), including haemophili, living mainly in a diverse community of biofilm [17–19]. Biofilm as a structure of microbial community enveloped in a polymeric matrix and adhered to both natural and synthetic surfaces may be regarded as a phenotypic adaptation and protective or pathogenic factor in many infections, depending on the condition [11, 20–22]. It was found to be a form of microbial life important both in colonization and in chronic and recurrent or acute diseases such as otitis media and pneumonia caused by NTHi species [17]. Biofilm is estimated to be involved in about 65 % of human infections with bacterial etiology [23]. Adhesive properties, as well as biofilm formation by microoorganisms together with its intrinsic antimicrobial resistance, exopolysaccharide production and *quorum sensing* are factors allowing for adaptation to host organism [20]. Both *H. influenzae* and *H. parainfluenzae* have been found to be a biofilm-forming bacteria.

The objectives of the present study were: the analysis of the correlations of diagnostic results in patients with sarcoidosis based on simple regression, haemophili isolation in nasopharyngeal and sputum specimens, antimicrobial resistance determination in *H. influenzae* and *H. parainfluenzae* clinical isolates, biofilm production by clinical isolates of these species together with the analysis of its structure.

## Methods

### Patients

A group of 31 adult patients (average age 42.6 ± 13) with a suspicion of sarcoidosis who were diagnosed in 2011 at the Chair and Department of Thoracic Surgery (Medical University of Lublin, Poland), participated in the study. The selection criterion was sarcoidosis, which was diagnosed with clinical findings suggesting an incidence of this disease. Patients were directed for diagnosis because of radiological findings such as: lymphadenectomy or tumour of mediastinum, or the presence of small nodules and infiltrations in the lung parenchyma, sclerosis, thickening or fibrosis discovered in CT scans. Multivariable demographic, clinical, radiographic and histological data were collected on the basis of the patients’ questionnaires and information protocol.

All patients were diagnosed by means of bronchoscopy, mediastinoscopy or/and lung biopsy. Before the procedure blood samples were collected for standard blood tests (basic metabolic panel and complete blood count). The obtained tissue samples were evaluated by the same pathomorphologist. The histopathological findings were usually described as tuberculosis like granulation which could be considered as sarcoidosis in accordance with clinical changes.

A control group of 37 healthy volunteers (average age 44.6 ± 11.6) who agreed to participate in the survey was also included. They did not suffer from respiratory infections and had not received an antimicrobial therapy for at least three months prior to the examination or had not been admitted to hospital for at least two years.

Written informed consent for participation was obtained from people who agreed to take part in the study and filled out the survey. The Ethics Committee of the Medical University of Lublin approved study protocol (KE-0254/75/2011).

### Microbiological processing of haemophili isolates

A total of 31 nasopharyngeal swabs and 31 sputum specimens were taken from patients with sarcoidosis on the day of hospitalization or a day after. Additionally, 37 nasopharyngeal specimens were collected from healthy people.

After incubation (48 h, 35 ± 2 °C, 5 % CO_2_) the colonies with morphological differences were identified independently on selective HAEM-medium (Haemophilus-chocolate-agar, bioMérieux, France). The growth of bacteria in the form of individual colonies or from abundant to very abundant number of morphologically different colonies on Chocolate agar was observed. Initially biochemical identification and biotyping of 192 Gram-negative isolates (125 – from patients with sarcoidosis and 67 – from healthy people) was carried out using the API-NH microtest (bioMérieux). The phenotypes of haemophili isolates were differentiated based on various observable properties in the growth morphology (e.g. the shape and size of the colony, smooth or rough surface, texture, colony elevation), on a set of biochemical reactions (according to API NH results) and antimicrobial susceptibility results. API-NH is a standardized system for the identification of *Neisseria*, *Haemophilus* (and related genera) and *Moraxella* (*B*.) *catarrhalis*, which uses microtests and a specially adapted database. Next, for the species differentiation the matrix-assisted laser desorption/ionization time-of-flight mass spectrometry (MALDI-TOF MS, Bruker Daltonik, Germany) system was used according to the procedure described earlier [24]. Software used for data acquisition was MALDI-Biotyper 3.0 (Bruker Daltonik) software. The species which were not identified as *H. influenzae* or *H. parainfluenzae* were classified as other *Haemophilus* spp.

Antibiotic sensitivities were determined by the disc diffusion method using Haemophilus-Test-Medium (HTM, Oxoid) according to [25]. Direct colony suspensions standardized to 0.5 McFarland standard were prepared using colonies from an overnight HAEM incubation (35 °C, 5 % CO_2_). *H. influenzae* ATCC10211 was used to verify the growth of bacteria on HTM medium. Different discs with antimicrobial agents (BD BBL™, Becton Dickinson and Company, USA) namely ampicillin 10 μg, amoxicillin/clavulanate 20/10 μg, ampicillin/sulbactam 10/10 μg, cefuroxime 30 μg, cefotaxime 30 μg, ceftazidime 30 μg, imipenem 10 μg, aztreoname 30 μg, azithromycin 15 μg, tetracycline 30 μg, trimethoprim/sulfamethoxazole 1.25/23.75 μg, ciprofloxacin 5 μg were used. Resistance (R) zone diameter interpretative criteria were ≤18 mm for ampicillin, ≤19 mm for amoxicillin-clavulanate and ampicillin-sulbactam, ≤16 mm for cefuroxime, ≤25 mm for cefotaxime and ceftazidime, ≤15 mm for imipenem, ≤ 25 mm for aztreoname, ≤11 mm for azithromycin, ≤25 mm for tetracycline, ≤10 mm for trimethoprim/sulfamethoxazole, ≤20 mm for ciprofloxacin, according to [25]. Multidrug-resistant (MDR) isolates were defined as having resistance to at least three different classes of antimicrobials. Beta-lactamase activity was screened by *Pen-*microtest (part of API-NH strip) and confirmed with a nitrocefin-based test (Cefinase™-Discs, BD BBL™) recommended for testing haemophili [25]. Then, E-test strips (bioMérieux) for a determination of susceptibility on the basis of the minimal inhibitory concentration of ampicillin (MIC_Am_) were used. The MIC criterion, defined as the lowest concentration of the antimicrobial agent that prevents visible growth of the microorganism, for ampicillin-resitant haemophili was ≥ 4 μg/ml [25].

### Biofilm detection

Biofilm formation was examined during the stationary culture *in vitro* in 24-well polystyrene microplates (24 F**-**Well Microplates, Thermo Scientific™ Nunc™, Denmark) using a 0.1 % crystal violet (CV) stain as previously described [26]. The Tripticasein Soy Broth (TSB, Biocorp, Poland) supplemented with Haemophilus Test-Medium Supplement (HTMS, Oxoid) designated as TSB + HTMS was used. Overnight cultures were diluted in TSB + HTMS-medium and standardized at 570 nm with an initial optical density of OD_570_ ~ 0.08 ± 0.02 (~0.5 McFarland-standard) using a microplate reader ELx800 (BioTek Inc., USA). Next, 500 μl of the microbial suspension was inoculated for each well and incubated (35 °C, 24 h, 5 % CO_2_). The growth of haemophili was assessed by measuring the OD_570_. Nonadherent cells were removed by rinsing the wells with sterile water. The biofilm was detected with OD_570_ according to the method based on staining with 500 μl 0.1 % CV. Each isolate was tested in triplicate in three series. TSB + HTMS without bacteria was incubated under the same conditions and served as blank control.

Haemophili were classified as biofilm-producers as described elsewhere [26]. The experiments were performed in triplicate and the results were averaged. For the purposes of a detailed analysis of the obtained results the classification of biofilm producers was introduced on the basis of criteria proposed by Stepanović et al. [27] and modified by Kosikowska et al. [26]. They defined the cut-off an optic density OD (OD_c_) for the microtiter-plate test as three standard deviations above the mean OD of the negative control. The bacteria were classified as follows: OD ≤ OD_c_ - non-producers (category 0); OD_c_ < OD ≤ 2 x OD_c_ - weak producers (category 1); 2 x OD_c_ < OD ≤ 4 x OD_c_ – moderate producers (category 2); 4 x OD_c_ < OD ≤ 8 x OD_c_ – strong producers (category 3), 8 x OD_c_ < OD – very strong producers (category 4). OD_c_ was 0.1 ± 0.02 in our experiments. Additionally, *H. influenzae* ATCC10211, *H. parainfluenzae* ATCC7901, *H. parainfluenzae* ATCC33392 and *H. parainfluenzae* ATCC51505 reference strains were used as biofilm producers. All tests were carried out three times and the results were averaged.

To visualize *H. influenzae* and *H. parainfluenzae* biofilm formation in a 24 h culture in 24-well polystyrene microplates, the inverted microscope Axiovert 200 M equipped with LSM5 Pascal Head (Carl Zeiss, Germany, with magnification 200×) was used. To obtain images of the biofilm, cultures were stained with Bacterial Live/Dead^®^*BacLight*™-L7012-kit (Invitrogen, USA) accordingly to the manufacturer’s procedure. Biofilm was stained with Live/Dead^®^ L7012 kit with two components, Syto9 and propidium iodide (PI), which are nucleic acid stains. The Syto9 stain penetrates through the intact and damaged cell wall, intercalates into DNA, and emits green fluorescence (detection of both live and dead bacteria). The PI stain diffuses only across the dead and damaged cell wall, intercalates into DNA and emits red fluorescence (detection of dead bacteria). The use of a combination of these two dyes is that PI displaces Syto9 (it had a higher affinity) reducing its fluorescence. Thus, the bacteria with intact cell membranes stain fluorescent green, whereas with damaged cell membranes stain fluorescent red. Biofilm formation by 7 *H. influenzae* and 18 *H. parainfluenzae* randomly selected clinical isolates taken in patients with sarcoidosis was screened using confocal microscopy. The thickness of biofilm (μm) and measurement area covered by biofilm (%), content of live cells (%) and biofilm area covered by live cells (%) were detected.

Overnight cultures were standardized in TSB + HTMS-medium. Then 500 μl/well of cultures was added (for each strain in quadruplicate). After incubation (35 °C, 24 h, 5 % CO_2_) the content of the wells was removed and each well was washed three-four times with 0.85 % NaCl. Then, 500 μl/well of 0.85 % NaCl was added and then stained with 1.5 μl of Live/Dead-kit solution. Microphotographs were taken in the green and/or red channel in a Confocal Laser Scanning Microscope LSM5-PASCAL (CLSM; Carl Zeiss, Germany) after incubation for 15 min in the dark at room temperature. This experiment was repeated twice.

The planimetric measurement of the biofilm was performed based on the microphotographs taken at 50× or 200× magnification in a two-dimensional scan (2D). Biofilm parameters were calculated using ImageJ-1.43e software (Wayne Rasband, National Institutes of Health, USA). A three-dimensional (3D) image of biofilm was reconstructed using the CLSM (200× magnification).

### Statistical analysis

The statistical analysis was performed with Statgraphics Plus for Windows, Version 4.1 (Statistical Graphics Corp. 1999, Statpoint Technologies, Inc. Warrenton, Virginia, USA). To assess the relationship between the variables a simple regression analysis was used. The aim of the statistical analysis was preliminary determination of the relationship between individual factors and the dependent variables. The relationship between age/gender and different clinical manifestations and laboratory findings were studied. The hypotheses were raised: H_0_ - there is no association between age/gender and different clinical manifestations and laboratory findings, and H_1_ - there is an association between age/gender and different clinical manifestations and laboratory findings. Quantitative variables were presented as mean (± standard deviation, SD) and median values. In some studies Fisher’s test was evaluated. The level of *P <* 0.05 was usually considered as statistically significant.

## Results

### Characteristics of patients

The main characteristics of patients with sarcoidosis are summarized in Table 1. Demographic and clinical data were obtained from the patients’ files and based on a questionnaire conducted for the presented variables. “Fatigue” was self reported as being unable to go any further. Susceptibility to “fatigue” was associated with a short walk on a flat surface, or with stair walk to the first or second floors. Symptoms such as shortness of breath, wheezing, cough, expectoration, hypertension, coronary artery disease, allergy, asthma, and recurrent infections (≥3/year) were also considered. Almost 26 % of the patients with sarcoidosis showed recurrent infections in childhood and had other symptoms such as anaemia, celiac disease, or endocrinological disorders. Some of them had two parallel disorders, for example hypertension and coronary artery disease (about 13 %), and/or allergy (about 10 %).

**Table 1:** Baseline clinical characteristics, laboratory findings, and predisposing factors in the group of patients with sarcoidosis

Variable	No. of cases/No. of patients or range	(%) or average ± SD
Gender		
Female	13/31	41.9
Male	18/31	58.1
Age (years)	24–64	42.6 ± 13.0
Tobacco smoke		
Active	9/31	29.0
Secondary	20/31	64.5
Area of residence		
Rural	15/31	48.4
Urban	16/31	51.6
Healthy condition		
Shortness of breath, wheezing	12/31	38.7
Cough	16/31	51.6
Expectoration	4/31	12.9
Fatigue:	22/31	71.0
a short walk (*n =* 1)	1/22	4.5
1^st^ floor (*n =* 4)	4/22	18.2
2^nd^ floor (*n =* 17)	17/22	77.3
Hypertension, coronary artery disease	4/31	12.9
Allergy	3/31	9.7
Asthma	1/31	3.2
Recurrent infection (≥3/year)	1/31	3.2
Recurrent infections in childhood	8/31	25.8
Other (anaemia, celiac disease, thyroid disorders)	8/31	25.8
Tuberculosis	2/31	6.5
Laboratory findings		
FVC (ml)	2740–6200	4208.1 ± 883.2
FEV_1_ (ml)	1600–5050	3231 ± 810.3
FEV_1_/FVC%	58–95	76.5 ± 8.7
pH	7.35–7.49	7.43 ± 0.03
pO_2_ (mmHg)	63.2–108	82.6 ± 10.5
pCO_2_ (mmHg)	32.8–49.1	95.4 ± 2.7
Saturation (%)	90.9–97.3	95.4 ± 1.8
IgG (g/l, reference: 7.0–16.0)	7.1–21.6	10.8 ± 2.7
IgM (g/l, reference: 0.4–2.3)	0.28–3.54	1.38 ± 0.8
IgA (g/l, reference: 0.7–4.0)	0.51–6.9	2.57 ± 1.3
CRP (mg/l, reference: 0.0–5.0):	0.51–80.48	7.23 ± 16.7
0.0–5.0 (*n =* 25)	0.51–4.15	1.51 ± 0.93
5.1–80.48 (*n =* 6)	5.8–80.48	31.10 ± 28.83
Changes in CT scan	30/30	100
Changes in X-ray	19/20	95
Changes observed during bronchofiberoscopy	14/17	82.4

Data were presented as: No. of positive samples/No. of examinations or value range; mean ± SD

*Abbreviations*: *CT* computed tomography, *CRP* C-reactive protein, *FEV*_*1*_ forced expiratory volume in one second, *FVC* forced vital capacity, *IgA, IgM, IgG* immunoglobulin A, M, G, *NP* nasopharynx, *SP* sputum; susceptibility to “fatigue” was associated with a short walk on a flat surface, or with stair walk to the first or second floors. “Fatigue” was self reported as being unable to go any further

There was a positive correlation (*P <* 0.1) between gender and a tumor of the lung/mediastinum (c = 0.4) or fluid in the pleural cavity (c = 0.32) in patients with sarcoidosis (Table 2). These changes mostly occurred in males rather than in females. A positive correlation was demonstrated between gender and CRP (C-reactive protein; *P =* 0.01, c = 0.44), and in elderly patients between the disease and visible changes (e.g. sclerosis, thickening or fibrosis) in the imaging diagnostics of the chest (*P <* 0.1, c = 0.34–0.45). A negative correlation between age and IgM (*P <* 0.1, c = -0.4) values was detected. Besides, changes in levels of IgG and IgA immunoglobulins were observed.

**Table 2:** Patient characteristics by age and gender correlations with clinical conditions, laboratory findings, and complications

Tests	Test results	Age (years)		Gender	
		*P* value	Correlation	*P* value	Correlation
Clinical manifestation	Sclerotic changes in lung	0.84	0.04	0.64	0.09
	Enlargement of the mediastinal lymph nodes	0.45	−0.14	0.9	0.02
	Tuberculosis like findings	0.23	0.23	0.23	0.23
Chest radiography	Micronodules	0.81	−0.05	0.63	0.12
	Pulmonary shading/infiltrations	0.05	0.45*	0.28	−0.26
	Tumor of the lung/mediastinum	0.36	0.22	0.08	0.4*
Chest CT	Changes sclerosis/thickening/fibrosis	0.61	0.22	0.64	0.04
	Lymphadenopathy	0.06	0.34*	0.16	−0.27
	Fluid in the pleural cavity	0.44	0.15	0.09	0.32*
Changes observed during bronchofiberoscopy	Infiltration of bronchial wall/macrophages	0.38	0.23	0.66	−0.12
	Scraps of bronchial epithelial/mucus	0.28	0.28	0.39	0.22
	Easy bleeding capillaries	0.17	−0.35	0.33	0.25
Laboratory findings	pH	0.77	−0.06	0.68	−0.08
	IgG (g/l)	0.74	−0.06	0.41	0.15
	IgM (g/l)	0.25	−0.4*	0.27	−0.2
	IgA (g/l)	0.17	0.25	0.47	0.14
	CRP (mg/l)	0.21	0.23	0.01	0.44**

*Abbreviations*: *CT* computed tomography, *CRP* C-reactive protein

The significance levels were marked as: *P < 0.1, and **P < 0.05)

### The prevalence of haemophili-positive clinical samples

**Fig. 1:**
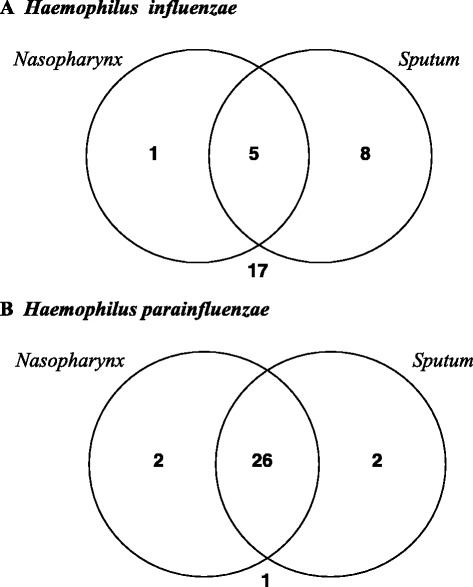
The prevalence of *Haemophilus influenzae* (**a**) and *Haemophilus parainfluenzae* (**b**) positive clinical samples in patients with sarcoidosis

In total, 30/31 (96.8 %) patients with sarcoidosis were colonized by *H. influenzae* and/or *H. parainfluenzae. H. influenzae* was isolated in 14/30 (46.7 %) patients - in 1/14 (7.1 %) only in nasopharynx, in 8/14 (57.1 %) only in the sputum and in 5/14 (35.7 %) patients in both samples (Fig. 1a). *H. parainfluenzae* was isolated in 30/30 (100 %) patients - in 2/30 (6.7 %) only in nasopharynx, in 2/30 (6.7 %) only in the sputum and in 26/30 (86.6 %) cases in both samples (Fig. 1b). The co-existence of *H. influenzae* and *H. parainfluenzae* was found in 14/30 (46.7 %) patients.

In comparison, nasopharynx in 31/37 (83.8 %) of healthy people was colonized by both species: in 1/31 (3.2 %) - by *H. influenzae*, in 31/31 (100 %) - by *H. parainfluenzae*. There were significant differences in the frequency of nasopharynx colonization by *H. influenzae* (*P =* 0.05), but not by *H. parainfluenzae* (*P =* 0.49) in patients with sarcoidosis and in healthy people.

Negative correlations (a simple regression) were found between *H. influenzae* presence in the sputum and O_2_ saturation (*P <* 0.01, c = -0.52) or easily bleeding capillaries (*P <* 0.05, c = -0.53), and between *H. influenzae* presence both in the nasopharynx and in the sputum and the sclerotic changes in lung (*P <* 0.05, c = -0.4 and c = -0.44, respectively). Positive correlations were found between *H. parainfluenzae* isolation in the sputum and the presence of fluid in the pleural cavity (*P <* 0.01, c = 0.56), pH value (*P <* 0.05, c = 0.39), or O_2_ saturation (*P <* 0.1, c = 0.32) and tuberculosis like findings (*P <* 0.1, c = 0.35). Negative correlations were found between *H. parainfluenzae* isolation in the sputum and FVC (Forced Vital Capacity; *P <* 0.1, c = -0.35).

### Number of haemophili isolates

From one to six different phenotypes of haemophili were isolated in the nasopharynx and/or in the sputum samples in each single patient. The growth morphology and biochemical features distinguished phenotypes of isolates. In total, 125 haemophili isolates were found in 62 nasopharyngeal swabs and sputum samples obtained in patients with sarcoidosis. In 30 haemophili-positive nasopharyngeal samples 59/125 (47.2 %) isolates were identified: 6/59 (10.2 %) as *H. influenzae*, 47/59 (79.7 %) as *H. parainfluenzae* and 6/59 (10.2 %) as other *Haemophilus* spp. In 30 haemophili-positive samples taken in the sputum, 66/125 (52.8 %) isolates were identified: 13/66 (19.7 %) as *H. influenzae*, 49/66 (74.2 %) as *H. parainfluenzae* and 4/66 (6.1 %) as other *Haemophilus* spp.

Likewise, in 31 haemophili-positive samples obtained in the nasopharynx of healthy people 67 isolates were identified: 1/67 (1.5 %) as *H. influenzae*, 65/67 (97.0 %) as *H. parainfluenzae*, and 1/67 (1.5 %) as other *Haemophilus* spp. *H. parainfluenzae* was the main species isolated from the nasopharynx in both groups. There were statistically significant differences between the number of *H. influenzae* (*P =* 0.05) or *H. parainfluenzae* (*P =* 0.0032) isolates in patients with sarcoidosis and in healthy people.

### Biotypes of *H. influenzae* and *H. parainfluenzae* isolates

A total of 19 *H. influenzae* and 96 *H. parainfluenzae* strains isolated from patients with sarcoidosis were assigned to eight biotypes. Biotypes III and VI occurred in 47.4 % and 26.3 % *H. influenzae* isolates respectively, mainly in the sputum (Table 3). Biotypes I (60.4 %) and II (30.2 %) constituted most of the *H. parainfluenzae* isolates both in the nasopharynx and in the sputum. For comparison, thedifferences were observed in nasopharyngeal *H. parainfluenzae* biotypes I-IV identified in healthy people and in patients with sarcoidosis (biotype I: 58.5 % vs. 26 %, *P =* 0.6998; biotype II: 20 % vs. 16 %, *P =* 0.1864; biotype III: 12.3 % vs. 5.2 %, *P =* 1.000 and biotype IV: 6.2 % vs. 1 %, *P =* 0.397, respectively).

**Table 3:** Distribution of *Haemophilus influenzae* and *Haemophilus parainfluenzae* biotypes in healthy people and in patients with sarcoidosis

	Healthy people	Patients with sarcoidosis		
Biotype	Nasopharynx	Nasopharynx	Sputum	Total
	No. (%) of isolates			
*Haemophilus influenzae*	*n =* 1		*n =* 19	
II	0 (0)	3 (15.8)	0 (0)	3 (15.8)
III	0 (0)	2 (10.5)	7 (36.8)	9 (47.4)
VI	1 (100)	1 (5.3)	4 (21.1)	5 (26.3)
VIII	0 (0)	0 (0)	2 (10.5)	2 (10.5)
*Haemophilus parainfluenzae*	*n =* 65		*n =* 96	
I	38 (58.5)	25 (26.0)	33 (34.4)	58 (60.4)
II	13 (20.0)	15 (15.6)	14 (14.6)	29 (30.2)
III	8 (12.3)	5 (5.2)	1 (1.04)	6 (6.3)
IV	4 (6.2)	1 (1.04)	0 (0)	1 (1.04)
VI	1 (1.5)	1 (1.04)	0 (0)	1 (1.04)
VII	0 (0)	0 (0)	1 (1.04)	1 (1.04)
VIII	1 (1.5)	0 (0)	0 (0)	0 (0)

### Antimicrobials sensitivity of *H. influenzae* and *H. parainfluenzae* isolates

As shown in Table 4, only 4/19 (21.1 %) *H. influenzae* isolates from patients with sarcoidosis were resistant to the antimicrobials - 2/19 (10.5 %) were trimethoprim/sulfamethoxazole-resistant and 2/19 (10.5 %) were tetracycline-resistant (Table 4). In contrast, 53/96 (55.2 %) *H. parainfluenzae* isolates were resistant to the antimicrobials – 18/96 (18.8 %) were trimethoprim/sulfamethoxazole-resistant and 15/96 (15.6 %) were tetracycline-resistant. In addition, 20/96 (20.9 %) *H. parainfluenzae* isolates were resistant to beta-lactams, including 8/96 (8.3 %) ampicillin-resistant ones.

**Table 4:** Distribution of antimicrobial resistance in *Haemophilus influenzae* and *Haemophilus parainfluenzae* isolates in healthy people and in patients with sarcoidosis

		No. (%) of resistant isolates						
Species	Place of isolation	Sxt	Te	Ctx	Caz	Sam	AmC	Am
Healthy people								
*Haemophilus influenzae* (*n =* 1)	Nasopharynx (*n =* 1)	0	0	0	0	0	0	0
*Haemophilus parainfluenzae* (*n =* 65)	Nasopharynx (*n =* 65)	8 (12.3)	4 (6.2)	4 (6.2)	5 (7.7)	3 (4.6)	1 (1.5)	10 (15.4)
Patients with sarcoidosis								
*Haemophilus influenzae* (*n =* 19)	Nasopharynx (*n =* 6)	1 (16.7)	0	0	0	0	1 (16.7)	0
	Sputum (*n =* 13)	1 (7.7)	2 (15.4)	0	0	0	0	0
*Haemophilus parainfluenzae* (*n =* 96)	Nasopharynx (*n =* 47)	10 (21.3)	9 (19.1)	2 (4.3)	3 (6.4)	0	0	6 (12.8)
	Sputum (*n =* 49)	8 (16.3)	6 (12.2)	1 (2.0)	5 (10.2)	0	0	2 (4.1)

*Abbreviations*: *Am* ampicillin, *AmC* amoxycillin/clavulanate, *Caz* ceftazidime, *Ctx* cefotaxime, *Sam* ampicillin-sulbactam, *Sxt* trimethoprim/sulfametoxazole, *Te* tetracycline

The presence of nasopharyngeal *H. parainfluenzae* isolates resistant to tetracycline (4/65; 6.2 %) and to trimethoprim/sulfametoxazole (8/65; 12.3 %) was shown in healthy people. In addition, 23/65 (35.4 %) isolates were resistant to beta-lactams, including 10/65 (15.4 %) ampicillin-resistant ones.

The difference in presence of nasopharyngeal *H. parainfluenzae* isolates resistant to tetracycline in patients with sarcoidosis and in healthy people (19.1 % vs. 6.2 %, *P =* 0.041) was statistically significant. The differences in resistance to trimethoprim/sulfametoxazole (21.3 % vs. 12.3 %, *P =* 0.297) and to beta-lactams (23.4 % vs. 35.3 %, *P =* 0.214), including ampicillin (12.8 % vs. 15.4 %, *P =* 1.000) were also found, but they were not statistically significant.

The ampicillin-resistant isolates selected in patients with sarcoidosis (MIC_Am_ ≥ 6 μg/ml) were beta-lactamase-positive, and were found within I and III biotypes (Table 5). High MICs for ampicillin were detected in two isolates taken in the nasopharynx (MIC = 128 and >256 μg/ml), and in one isolate taken in the sputum (MIC = 48 μg/ml). Among *H. parainfluenzae* isolates, 3/96 (3.1 %) isolates were resistant to beta-lactams, tetracycline, and trimethoprim/sulfametoxazole (MDR-strains).

**Table 5:** Distribution of beta-lactamase positive ampicillin-resistant *Haemophilus parainfluenzae* isolates in patients with sarcoidosis

Biotype	Nasopharynx		Sputum	
	Resistance profile (No. of isolates)	MIC_Am_ (μg/ml)	Resistance profile (No. of isolates)	MIC_Am_ (μg/ml)
I	Am (1)	24	AmTe (1)	4
	AmSxt (1)	>256	AmTe (1)	48
	AmCtxCazSxt (1)	8		
	AmTeSxt (1)	128		
III	AmCtxCazTeSxt (1)	6		
	AmTeSxt (1)	6		

*Abbreviations*: *Am* ampicillin, *Caz* ceftazidime, *Ctx* cefotaxime, *Te* tetracycline, *Sxt* trimethoprim/sulfametoxazole

Ampicillin breakpoints used for interpretation of minimal inhibitory concentration (MIC_Am_): susceptible ≤1 μg/ml, intermediate = 2 μg/ml and resistant ≥ 4 μg/ml, according to [25]

### Biofilm formation by *H. influenzae* and *H. parainfluenzae* isolates

In total, 69 randomly selected *H. influenzae* and *H. parainfluenzae* isolates (35 - nasopharyngeal, 34 - from the sputum) taken in patients with sarcoidosis were screened for biofilm-production using the CV method. According to Table 6, 8/12 (66.7 %) isolates of *H. influenzae* and 39/57 (68.4 %) of *H. parainfluenzae* were found to be biofilm-producers (*P =* 0.407). All 8/12 (66.7 %) *H. influenzae* isolates were weak-producers, irrespective of the clinical specimens. Among 19 biofilm-forming *H. parainfluenzae* isolates from nasopharynx, the ability to biofilm formation ranged from weak (11/19, 57.8 %) to strong (1/19, 5.3 %) and very strong (7/19, 36.8 %).

**Table 6:** Distribution of *Haemophilus influenzae* and *Haemophilus parainfluenzae* biofilm-producers in patients with sarcoidosis and in healthy people

Category of biofilm producers	OD_570_ range	No. (%) of isolates			
		*Haemophilus influenzae*		*Haemophilus parainfluenzae*	
Healthy people					
		Nasopharynx (*n =* 1)		Nasopharynx (*n =* 65)	
Non-producers	0.0	0 (0.0)		8 (12.3)	
Weak	0.01–0.24	1 (100)		15 (23.1)	
Moderate	0.25–0.48	0 (0.0)		3 (4.6)	
Strong	0.49–0.96	0 (0.0)		12 (18.5)	
Very strong	≥0.97	0 (0.0)		27 (41.5)	
Patients with sarcoidosis					
		Nasopharynx (*n =* 4)	Sputum (*n =* 8)	Nasopharynx (*n =* 31)	Sputum (*n =* 26)
Non-producers	0.0	1 (25.0)	3 (37.5)	12 (38.7)	6 (23.1)
Weak	0.01–0.24	3 (75.0)	5 (62.5)	11 (35.5)	9 (34.6)
Moderate	0.25–0.48	0 (0.0)	0 (0.0)	0 (0.0)	3 (11.5)
Strong	0.49–0.96	0 (0.0)	0 (0.0)	1 (3.2)	4 (15.4)
Very strong	≥0.97	0 (0.0)	0 (0.0)	7 (22.6)	4 (15.4)

Distribution of biofilm-producers was detected using the crystal-violet (CV) method. The cut-off OD (OD_c_; here: 0.1 ± 0.02) was defined as three standard deviations above the mean OD_570_ of the negative control. The categories of biofilm-producers: 0 - non-producers (OD ≤ OD_c_); 1, weak (OD_c_ < OD ≤ 2xOD_c_); 2, moderate (2xOD_c_ < OD ≤ 4xOD_c_); 3, strong (4xOD_c_ < OD ≤ 8xOD_c_); 4, very strong (8xOD_c_ < OD) producers, according to [26]

Additionally, reference strains were included as biofilm-producers: *H. influenzae* ATCC10211as weak biofilm-producer (OD_570_ = 0.098 ± 0.05), *H. parainfluenzae* ATCC7901 as strong biofilm-producer (OD_570_ = 0.682 ± 0.07), and both *H. parainfluenzae* ATCC33392 and *H. parainfluenzae* ATCC51505 as very strong biofilm-producers (OD_570_ = 1.191 ± 0.04 and OD_570_ = 1.026 ± 0.08, respectively).

In 65 *H. parainfluenzae* isolates obtained from nasopharynx of healthy people, in 57/65 (87.7 %) isolates the ability for biofilm formation was identified (Table 6). Among 57 biofilm-positive *H. parainfluenzae* isolates 15/57 (26.3 %), 3/57 (5.3 %), 12/57 (21.1 %), and 27/57 (47.4 %) ones were classified as weak, moderate, strong or very strong biofilm-producers, respectively. Significant differences between the ability to biofilm production in nasopharyngeal *H. parainfluenzae* isolates taken in patients with sarcoidosis and in healthy people were shown (*P =* 0.006). Among biofilm-positive *H. parainfluenzae* isolates taken in cases and in healthy people significant differences were observed between weak biofilm-producers and the group of isolates classified as moderate, strong and very strong biofilm-producers (*P =* 0.024).

Morphometric parameters of biofilm formed by 7 *H. influenzae* and 18 *H. parainfluenzae* isolates taken from the patients with sarcoidosis were assessed by means of CLSM technique (Table 7). The biofilm formed by *H. influenzae* isolates had the thickness of 14.2 ± 3.5 μm and covered 71.6 ± 5.6 % of the measured area. The content of living cells was from 8.6 to 95.2 % (average 71.6 ± 4.4 %) and it was 75.2 ± 4.8 % in the biofilm area. The biofilm formed by *H. parainfluenzae* isolates had the thickness of 20.02 ± 4.3 μm and covered about 50.6 ± 4.8 % of the measured area. The content of living cells was from 42.8 to 99.9 % (average 80.9 ± 4.8 %) and it was about 77.9 ± 8.98 % in the biofilm area.

**Table 7:** Morphometric parameters of biofilm formed by *Haemophilus influenzae* and *Haemophilus parainfluenzae* isolates taken from patients with sarcoidosis

Haemophili isolates selected from	Thickness of biofilm ± SD (μm)	Measurement area covered by biofilm (%) ± SD	Content of live cells (%) in biofilm ± SD	Biofilm area covered by live cells (%) ± SD
	*Haemophilus influenzae*			
Nasopharynx (*n =* 3)	11.3 ± 3.2	68.9 ± 2.5	58.4 ± 2.97	66.99 ± 9.1
Sputum (*n =* 4)	16.4 ± 3.7	73.6 ± 7.96	81.5 ± 5.4	81.4 ± 1.6
All (*n =* 7)	14.2 ± 3.5	71.6 ± 5.6	71.6 ± 4.4	75.2 ± 4.8
	*Haemophilus parainfluenzae*			
Nasopharynx (*n =* 10)	20.5 ± 4.9	47.1 ± 5.9	76.8 ± 4.2	72.5 ± 12.5
Sputum (*n =* 8)	19.4 ± 3.7	54.95 ± 3.5	86.0 ± 5.4	84.6 ± 4.7
Total (*n =* 18)	20.0 ± 4.3	50.58 ± 4.8	80.9 ± 4.8	77.9 ± 8.98

Averages morphometric parameters of biofilm formed by *Haemophilus influenzae* and *Haemophilus parainfluenzae* isolates was calculated on the basis of data assessed using confocal laser scanning microscopy (CLSM) images after 24 h incubation

**Fig. 2:**
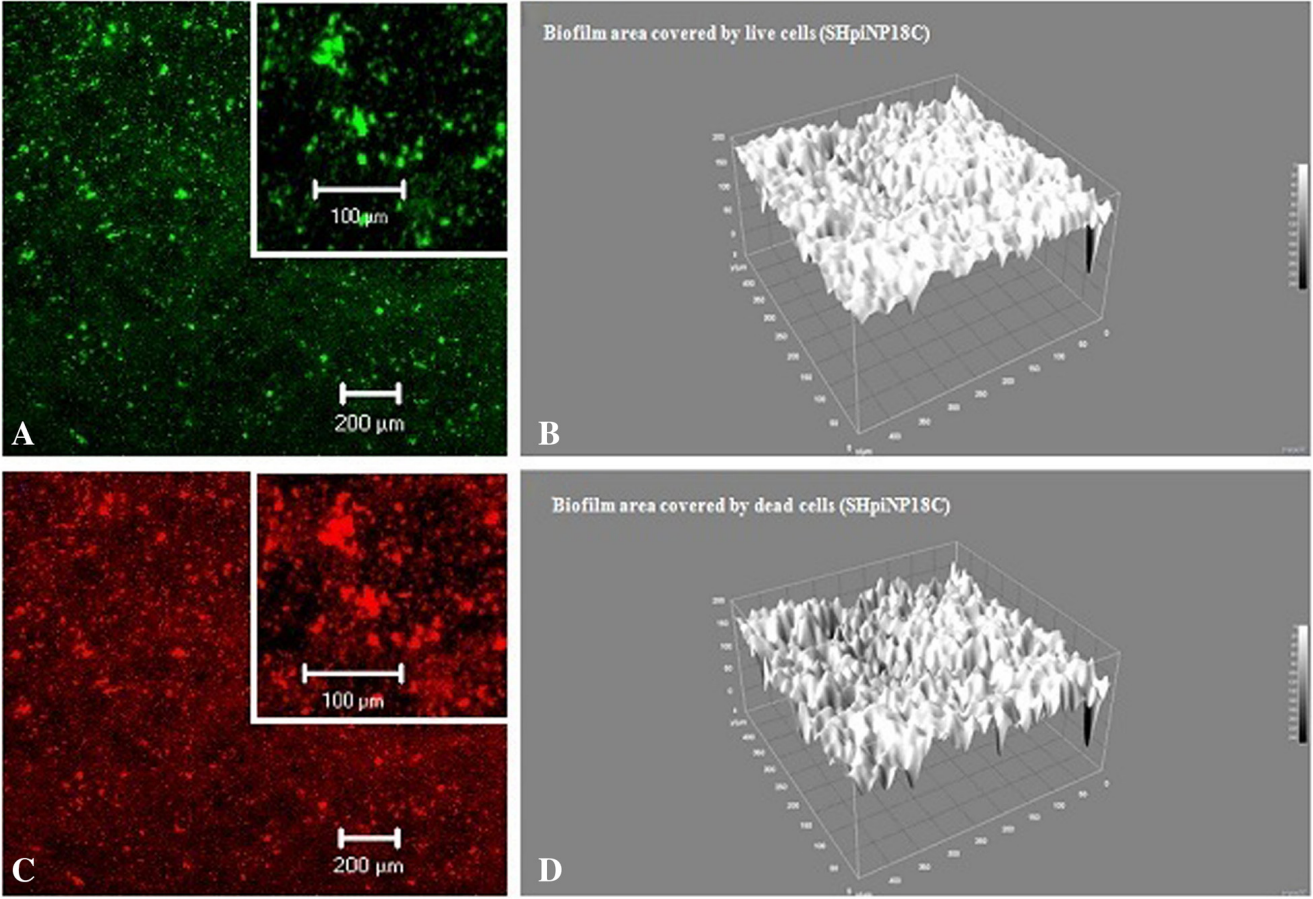
Biofilm formed by living and dead cells of *Haemophilus parainfluenzae* SHpiNP18C strain. Expanations: Biofilm formed by living (**a**, **b**) and dead (**c**, **d**) cells of *Haemophilus parainfluenzae* SHpiNP18C and detected using two-dimensional (2*d*: **a**, **c**) or three-dimensional (3*d*: **b**, **d**) CLSM image with gray level values varying from 0 to 250 (**b**, **d**) after 24 h of incubation. The layer of living cells was located on or between the structures formed by dead cells. The living and dead cells forming biofilm were detected in the green and red canals, respectively, and the structure of the biofilm was shown in XZ plan on the basis of 3D scan

The biofilm formed by one of *H. parainfluenzae* nasopharyngeal isolate taken from a patient with sarcoidosis was revealed by CLSM image (Fig. 2). The biofilm area formed by living (Fig. 2a) and dead (Fig. 2c) cells is presented. The textural parameters were detected as the grey scale intensity of the biofilm formed by live (Fig. 2b) and dead (Fig. 2d) cells.

## Discussion

Several environmental agents interacting with social and genetic factors and including immune responses to microbial components rather than an infection *per se* have been considered to play a role in the pathogenesis of sarcoidosis [1, 2, 4]. In our study changes in the healthy conditions, in the imaging diagnostics of the chest and in levels of three major immunoglobulins (IgG, IgM and IgA) as well as CRP value with respect to sarcoidosis and the age and gender were shown (Tables 1 and 2). Similar observations were also done by other authors [28–31]. Hiperglobulinemia is frequently observed in patients with sarcoidosis [30]. Changes observed in this group of patients [29, 30] and in elderly people [31] suggested an association between age, race, sex and the immune system’s defense as well as microbials presence. According to Buckley et al. [29], in patients with sarcoidosis the increase in IgG was significant in white patients, and the increase in IgM concentration was significant only in black patients, especially in black woman. Cagatay et al. [32] found higher than normal laboratory values for immunoglobulins IgG, IgA and IgM. They have noted that IgG and IgA levels were significantly higher in the group of routinely checked patients without any clinical symptoms and during established the activity of the disease. Drent et al. [33] have identified a high CRP concentration associated with severe fatigue in sarcoidosis.

The human microbiota has multidirectional effects on the host’s health and changes in its composition may have important consequences for human pathophysiology and disease development [5, 7, 8, 34]. According to literature, bacterial 16S rRNA from *H. influenzae* as well as *Moraxella catarrhalis* was detected in sarcoid fluid samples [35]. Cantwell’s review [3] pointed to the presence of other bacterial species (e.g. *Mycobacterium* spp., staphylococci, *Propionibacterium acnes*) in the biopsy of sarcoid tissue, blood, and skin samples taken from patients with sarcoidosis.

According to our results, higher rate of nasopharyngeal colonization by *H. influenzae*, but not by *H. parainfluenzae* (*P =* 0.05 vs. *P =* 0.49) was found in patients with sarcoidosis as compared to colonization in healthy people. It suggests that sarcoidosis can be regarded as a factor predisposing for colonization by *H. influenzae*. Besides, *H. influenzae* was frequently found in the sputum samples taken in patients with sarcoidosis even if this bacterial species was absent in the nasopharynx. In our opinion, it suggests the role of *H. influenzae* in the lower respiratory tract colonization and inflammation particularly in chronic diseases, e.g. in patients with sarcoidosis. In the literature [36], it has been documented that a combination of pathogenic mechanisms of bacteria and defects in host defense may allow this species to their migration into the lower respiratory tract. This may result in chronic colonization and/or in acute exacerbations of airway disease.

Despite the high and a similar number of people colonized by *H. parainfluenzae* in the nasopharynx of healthy people and patients with sarcoidosis (83.8 % vs. 96.8 %), we observed a decreased number of this species isolates with different phenotypes, differentiated on the basis of growth morphology, biochemical characteristics and biotypes. In 30 patients with sarcoidosis 47 isolates of *H. parainfluenzae* were identified, while in 31 healthy people - 65 isolates of this species. *H. parainfluenzae* isolates with biotypes I and II were found to occur most frequently, similarly in patients with sarcoidosis and in healthy people (Table 3). As found by other authors [37–39], these biotypes constituted most of *H. parainfluenzae* isolates in patients with other respiratory diseases such as chronic bronchitis or cystic fibrosis.

During our studies we compared the antimicrobial susceptibility of nasopharyngeal *H. parainfluenzae* isolates obtained in patients with sarcoidosis and in healthy people (Table 4). Resistance of these isolates to tetracycline (*P =* 0.041) and trimethoprim/sulfametoxazole (*P =* 0.297) in patients with sarcoidosis was higher compared to that in healthy people. In contrast, resistance to beta-lactams (*P =* 0.214), including ampicillin (*P =* 1.000) was lower in patients with sarcoidosis compared to that in healthy people. These differences may be due to higher consumption of a given group of antimicrobials in a defined population. The growing antibiotic resistance and reduction or elimination their effectiveness is one of the world’s most pressing public health problems [40].

Beta-lactam antibiotics are the most widely used antimicrobial agents during treatment of both community-acquired and hospital infections. The resistance to this group of antimicrobials in *Haemophilus* spp. usually is mediated by the production of beta-lactamases and the presence of altered penicillin-binding protein (PBP) with lowered affinity for these antibiotics as a target site [17, 41]. The ampicillin-resistant, beta-lactamase positive *H. parainfluenzae* isolates from patients with sarcoidosis were found in our studies (Tables 4 and 5). This may have some implications including the possibility to exchange resistance genes within microorganisms [40–45].

According to literature, over the past years many authors detected beta-lactamases mainly in *H. influenzae* and rarely in *H. parainfluenzae* isolates taken from patients with respiratory tract infections as well as from healthy people [25, 26, 46]. It was shown that DNA mutation and rapid multiplication as well as transformation can be important mechanisms in the spread of drug resistance in haemophili, including the ampicillin resistance due to beta-lactamase production [45, 46]. It seems that especially efficient in transformation were *H. parainfluenzae* cells with a highest ability to develop competence and transfer of resistance genes occurs via free DNA (from dead or lysed cells) during natural transformation from the medium by competent cells. It may explain the acquisition of resistance or resistance gene exchange with other microorganisms. Besides, resistance and reduced susceptibility to beta-lactams mediated by altered PBPs is also important in many bacterial pathogens, including beta-lactamase negative *H. influenzae* [47]. For this reason, there are different events that may contribute to the emerge of resistance: the acquisition of resistance genes (e.g. beta-lactamases) by conjugation or transformation; and inter-species recombination of the *ftsI* gene [47, 48]. According to Gromkova et al. [46], most efficient in transformation among *H. parainfluenzae* strains was biotype II, followed by biotype I.

It was shown in this paper that *H. parainfluenzae* isolates selected in patients with sarcoidosis compared to isolates selected in healthy people had a lower ability for biofilm production (Table 6). It is possible that a reduction in the number of *H. parainfluenzae* strains capable of biofilm formation may contribute to an increased colonization by certain opportunistic pathogens like *H. influenzae* (the present results) or by other bacteria [3, 4]. The CLSM technique revealed that biofilms formed *in vitro* by *H. parainfluenzae* and *H. influenzae* isolates taken from patients with sarcoidosis (Table 7, Fig. 2) had high content of live cells (average 72 to 81 %), suggesting the possibility of bacterial persistence and dispersal *in vivo*.

Biofilm may be regarded as a pathogenic or protective factor depending on the conditions [18, 21]. Nontypeable *H. influenzae* [NTHi] biofilms were observed for both bacteria colonizing the tissue of human, as an etiologic agent which causes the infection or exacerbation of chronic respiratory diseases [49–51]. On the other hand, Clancy and Dunkley [52] showed that oral NTHi could enhance the mucosal protection and prevent exacerbations of a chronic obstructive pulmonary disease.

On the basis of our results we propose, that haemophili, mainly *H. influenzae* and *H. parainfluenzae*, would be microorganisms indicative in respiratory microbiota changes as well as healthy condition in patients with chronic diseases. We observed higher frequency of *H. influenzae* colonization in patients with sarcoidosis compared to healthy people (*P =* 0.05). All *H. influenzae* isolates were weak- or non-biofilm producers independently to source of isolation and peoples’ health condition. Moreover, despite the high prevalence of *H. parainfluenzae* in the nasopharynx of people from both studied groups (*P =* 0.49), less number of isolates of this species obtained in nasopharyngeal samples in patients with sarcoidosis as compared to healthy people were classified as biofilm-producers (61.3 % vs. 87.7 %, *P =* 0.006), especially as strong and very strong biofilm-producers (25.8 % vs. 60 %, *P =* 0.002).

## Conclusions

The obtained results suggest that sarcoidosis, associated with many different factors, may be partially due to the respiratory microbiota condition. This is the first study describing qualitative and quantitative changes in the respiratory microbiota in patients with sarcoidosis with the respect to *H. influenzae* and *H. parainfluenzae* biotypes and their ability for biofilm formation. The question whether the biofilm formed by these bacterial species is a causative or just a protective factor in recurrent or chronic diseases, e.g. sarcoidosis, requires further studies.

## Abbreviations

Am: Ampicillin
AmC: Amoxycillin/clavulanate
Caz: Ceftazidime
CRP: C-reactive protein
CSLM: Confocal scanning laser microscopy
CT: Computed tomography
Ctx: Cefotaxime
CV: Crystal violet
FEV_1_: Forced Expiratory Volume/one second
FVC: Forced Vital Capacity
HAEM: *Haemophilus-*chocolate-agar
HTM: Haemophilus Test Medium
HTMS: Haemophilus-Test-Medium-Supplement
MALDI-TOF MS: Matrix-assisted laser desorption/ionization time-of-flight mass spectrometry
MDR: Multidrug-resistant
MIC: Minimal inhibitory concentration
NP: Nasopharynx
Sam: Ampicillin/sulbactam
SP: Sputum
Sxt: Trimethoprim/sulfametoxazole
Te: Tetracycline
TSB: Tripticasein-Soy-Broth
TSB + HTMS: Tripticasein-Soy-Broth supplemented with Haemophilus-Test-Medium-Supplement
